# Response of Bilayer and Trilayer Graphene to High-Energy Heavy Ion Irradiation

**DOI:** 10.3390/ma16041332

**Published:** 2023-02-04

**Authors:** Damjan Iveković, Sunil Kumar, Andrea Gajović, Tihana Čižmar, Marko Karlušić

**Affiliations:** Ruđer Bošković Institute, Bijenička Cesta 54, 10000 Zagreb, Croatia

**Keywords:** ion irradiation, graphene, Raman spectroscopy, defects

## Abstract

High-energy heavy ion irradiation is a very useful tool for the nanostructuring of 2D materials because defects can be introduced in a controlled way. This approach is especially attractive for the mass production of graphene nanomembranes when nanopore size and density can easily be tuned by ion irradiation parameters such as ion energy and applied fluence. Therefore, understanding the basic mechanisms in nanopore formation due to high-energy heavy ion impact is of the highest importance. In the present work, we used Raman spectroscopy to investigate the response of bilayer and trilayer graphene to this type of irradiation. Spectra obtained from graphene samples irradiated with 1.8 MeV I, 23 MeV I, 3 MeV Cu, 18 MeV Cu, and 12 MeV Si beams were analysed using the Lucchese model. It was found that the efficiency of damage production scales strongly with nuclear energy loss. Therefore, even for the most energetic 23 MeV I beam, the electronic energy loss does not contribute much to damage formation and ion tracks are unlikely to be formed.

## 1. Introduction

Graphene, a prominent representative of 2D materials, has attracted much attention with respect to defect engineering by ion irradiation. In particular, the perforation of graphene by high-energy heavy ion irradiation opened a new route for the production of graphene nanomembranes in large quantities [1,2,3,4]. However, the direct imaging of nanopores is challenging because they can be extremely small [4], so indirect methods for defect characterisation are preferred. In this context, Raman spectroscopy has proven to be extremely useful for the analysis of graphene since it allows for the measurement of the number of layers, doping, strain, and defect types and concentrations [5,6,7,8,9,10]. Therefore, great efforts have been made to understand the characteristics of Raman spectra with respect to the generation of damage in graphene via ion irradiation [9,10,11,12,13,14,15,16,17,18].

Bilayer (BLG) and trilayer graphene (TLG), often considered together as few-layer graphene, have also been recently investigated for their stability under ion irradiation [10,11,19,20,21,22,23,24,25]. Experimentally, their damage kinetics were found to be similar to that of single layer graphene (SLG), i.e., it is possible to describe it with the Lucchese model [8], though the size of defects in BLG and TLG appear to be somewhat smaller than in SLG [11,25]. However, the comprehensive investigation of damage formation in BLG and TLG exists only for low-energy ion irradiation [10,11,19,21,26], while there are just a few experimental studies on the high-energy ion irradiation of BLG and TLG [22,23,25,27]. Since the physical mechanisms of defect production could be quite different at low and high ion energies, it is important to determine which processes are relevant at high (i.e., MeV) ion energies.

Damage production using low energy heavy ion irradiation originates from close encounters between ions and the target atomic nuclei, i.e., the so-called nuclear energy loss, *dE_n_*/*dx*. Compared to bulk materials, where clusters of defects can be produced by a single ion impact, such clusters generated by recoil cascades are much smaller and rarer in 2D materials. This is particularly true for free-standing 2D materials, where recoils are simply sputtered away, so single atom defects such as vacancies are most often found [28]. At high kinetic energies, typically above 1 MeV/nucleon, the impact of the heavy ion leads to the heating of the electrons due to the electron energy loss *dE_e_*/*dx*, while nuclear collisions are very rare. Therefore, the defect formation occurs via the thermal spike scenario [16,29], where the amount of deposited energy is calculated (e.g., using the SRIM code [30]) under the assumption that the thickness of the SLG is 0.33 nm [16]. Heating of the material due to electron-phonon coupling could lead to the melting of the material, and as a consequence of the rapid quenching, permanent damage (called ion track) can occur. In some cases, sputtering of the material may also occur [31,32]. Recently, corrections have been made that consider energy dissipation from the 2D material via the emission of electrons, thus lowering the amount of deposited energy up to 50% [24,33,34]. Clearly, this should make 2D materials such as graphene quite resistant to electronic energy loss, and therefore, the threshold for the ion track formation in graphene should be investigated more closely.

The aim of the present work is to investigate experimentally the damage accumulation in supported BLG and TLG. By choosing suitable ion beams in terms of the ion type (iodine, copper, and silicon) and ion energy (1.8–23 MeV), a comparison of electronic and nuclear energy loss contributions was made. The obtained results provide information about damage production efficiency and the typical size of the damage and are relevant for applications such as nanomembranes, where BLG and TLG may be the preferable choice over SLG.

## 2. Experimental Details

Commercially available BLG and TLG samples (Graphenea, San Sebastian, Spain) were used in this work. The CVD-grown graphene 1 × 1 cm^2^ in size was deposited on a 300 nm thick a-SiO_2_ film covering a silicon wafer. Samples were not further processed before nor after the irradiation. 

High-energy heavy ion irradiation was performed using a 6 MV EN Tandem Van de Graaff accelerator (HVEE, Burlington, MA, USA) and the ToF-ERDA beamline located at the Ruđer Bošković Institute (Zagreb, Croatia) [35,36]. In Table 1, all ion beams used in the present work are listed. Ion irradiation parameters and damage evaluation were calculated using the SRIM code [30]. The ion beams were selected not only to cover a wide range of ion irradiation parameters but also to allow a direct comparison of the different energy loss mechanisms. All irradiations were performed at normal incidence. Previously, we have shown that the charge state of the ion plays a role in damage formation in SLG, while in the case of BLG and TLG, its contribution is minimal [25]. For this reason, we did not use stripping foil for charge state equilibration before the ion impact. During irradiation, the ion beam was scanned, and the irradiated area was determined to be 3 × 2 mm^2^ in size. The applied ion fluences were measured before and after exposures via ion flux monitoring. During high fluence irradiations, exposures were interrupted for additional flux measurements.

After irradiation, samples were investigated by Raman spectroscopy using the Horiba Jobin Yvon T64000 spectrometer, also located at the Ruđer Bošković Institute. A 532 nm solid-state laser with a 50× long working distance objective and laser power of a few mW was used.

## 3. Experimental Results and Discussion

**Figure 1:**
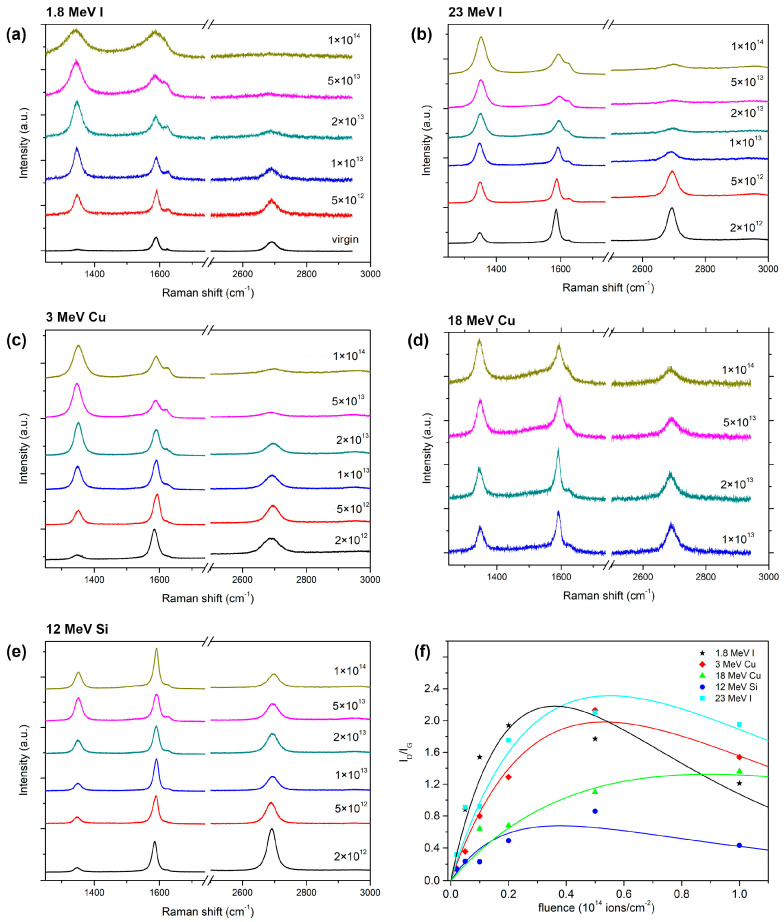
Raman spectra (532 nm excitation wavelength) of BLG samples irradiated with (**a**) 1.8 MeV I, (**b**) 23 MeV I, (**c**) 3 MeV Cu, (**d**) 18 MeV Cu, and (**e**) 12 MeV Si. Ion fluences (depicted by different colours and given in ions/cm^2^ units) are given above each spectrum. (**f**) Ratio *I*_D_/*I*_G_ for all used ion beams fitted using the Lucchese model (Equation (1)).

**Figure 2:**
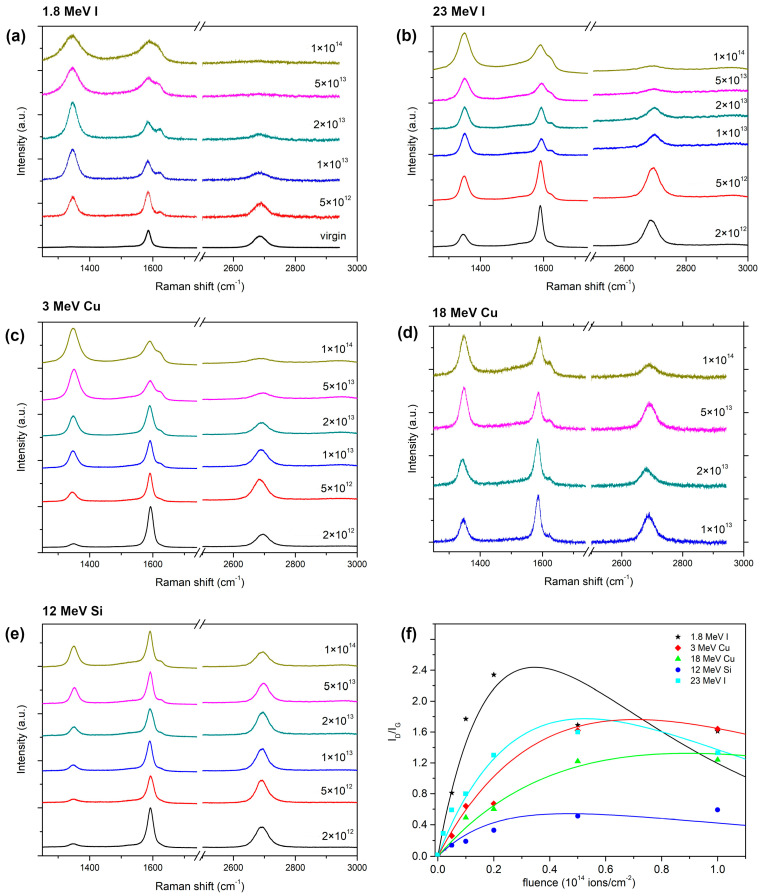
Raman spectra (532 nm excitation wavelength) of TLG samples irradiated with (**a**) 1.8 MeV I, (**b**) 23 MeV I, (**c**) 3 MeV Cu, (**d**) 18 MeV Cu, and (**e**) 12 MeV Si. Ion fluences (depicted by different colours and given in ions/cm^2^ units) are given above each spectrum. (**f**) Ratio *I*_D_/*I*_G_ for all used ion beams fitted using the Lucchese model (Equation (1)).

Raman spectroscopy measurements indicate the very good crystalline quality of the unirradiated samples. Two main peaks in the graphene spectrum, i.e., the G peak at 1580 cm^−1^ and the 2D peak at 2700 cm^−1^, are pronounced, while the peaks activated by defects (i.e., the D peak at 1350 cm^−1^ and the D′ peak at 1620 cm^−1^) are very small [25]. After the high-energy heavy ion impacts, defects are introduced into the graphene. As a result, the perfect hexagonal crystal lattice of the graphene is damaged and profound changes are found in the Raman spectra. All experimentally obtained Raman spectra of irradiated BLG and TLG samples are presented in Figure 1 and Figure 2, respectively. The spectra from unirradiated and 23 MeV I irradiated samples (within the 5 × 10^12^–5 × 10^13^ ions/cm^2^ fluence range) have already been reported [25].

Damage kinetics can be determined by observing the changes in the D peak. More precisely, the ratio of the D and G peaks intensities (*I_D_*/*I_G_*) is usually considered a measure of the disorder in graphene. When ion impact damage is non-overlapping, the *I_D_*/*I_G_* ratio increases linearly with the fluence. Then, it typically saturates at medium fluences when the ion impact damage begins to overlap. Finally, at the highest fluences, it begins to decrease as the graphene structure becomes amorphous (also seen as the disappearance of the 2D peak). The Lucchese phenomenological model (Equation (1)) captures this type of damage kinetics by considering two circular zones around the ion impact point: a structurally disordered region characterised by radius *r_s_*, and Raman active region characterised by the radius *r_a_*. Furthermore, the Lucchese model assumes that each ion impact produces a disordered region of identical circular shape. Most likely, such a circular shape is not the exact morphology of damage, as molecular dynamics illustrate convincingly [16], and thus *r_s_* should be considered only as an average measure of disordered material at the ion impact site. For example, each 90 eV Ar ion impact produces a disordered region with *r_s_* = 1 nm and an activated region with *r_a_* = 3 nm [8].

(1) $$\frac{I_{D}}{I_{G}}=C_{a}\frac{r_{a}^{2}-r_{s}^{2}}{r_{a}^{2}-2r_{s}^{2}}\left[exp\left(-\pi r_{s}^{2}F\right)-exp\left(-\pi \left(r_{a}^{2}-r_{s}^{2}\right)F\right)\right]+C_{s}\left[1-exp\left(-\pi r_{s}^{2}F\right)\right]$$

In the above equation, *F* is the fluence given in ions/nm^2^. The constants *C_a_* and *C_s_* represent the material response when the entire graphene surface is Raman active (*C_a_*) or structurally disordered (*C_s_*). For the 514 nm laser, these constants typically have values of *C_a_* = 4.5 and *C_s_* = 0. The Raman relaxation length for the resonant Raman scattering *L* in graphene for a 514 nm laser is 2 nm [8]. It establishes coupling between *r_s_* and *r_a_* (Equation (2)) which does not depend on the ion irradiation conditions.

(2) $$L=r_{a}-r_{s}=2\;\mathrm{nm}$$

In the later work, the probability *P* of damage production was introduced in the Lucchese model (Equation (1)), with the fluence *F* replaced by *PF* [14]. Afterwards, a similar approach could also be found in works by other groups [15,37]. Considering the Raman relaxation length for the resonant Raman scattering, *L* = 2 nm is fixed and does not depend on the irradiation parameters; the probability for defect formation *P* can be evaluated from this modified Lucchese equation [14,15]. This approach is important for sub-nanometre damage production, for example, near the ion track formation threshold. Under such irradiation conditions, it is reasonable to expect that different ion beams yield significant variance in damage production, both in terms of probability of damage production, as well as in damage morphology.

**Figure 3:**
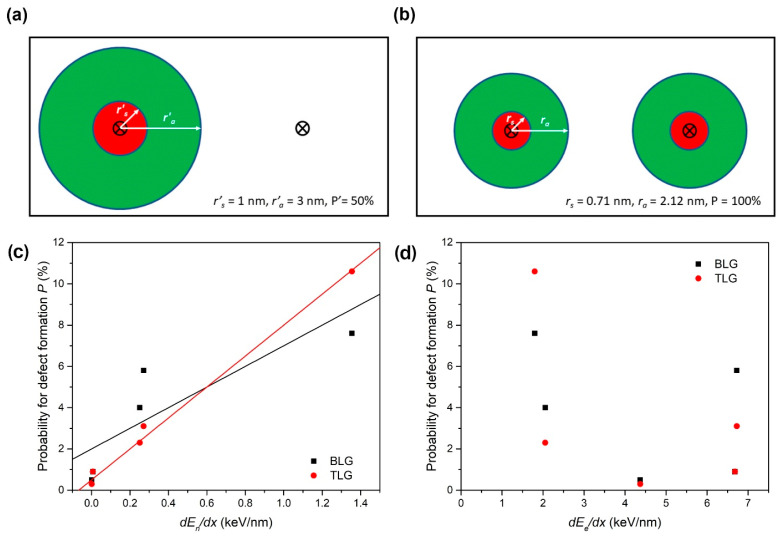
Illustration of the identical Raman response under the assumption of damage probability equal to (**a**) *P′* = 50% and (**b**) *P* = 100% (x marks the ion impact spot). Damage production probability *P* for BLG and TLG, correlated with (**c**) nuclear energy loss *dE_n_*/*dx* and (**d**) electronic energy loss *dE_e_*/*dx* (calculated by SRIM [30] for the graphite).

In the analysis of our spectra (after background subtraction and peaks fitting to the Lorentzian function), we apply a similar procedure that leads to essentially the same results as analysis using a modified Lucchese equation [14,15,37]. As an example, we show in Figure 3a a hypothetical scenario when 90 eV Ar ion impacts [8] produce typical damage (*r′_s_* = 1 nm, *r′_a_* = 3 nm), but with the probability of damage formation *P′* = 50%. An analysis of such an irradiated sample with the Lucchese model (Equation (1)), when every ion impact produces identical damage, would yield results *r_s_* = 0.71 nm, *r_a_* = 2.12 nm, and *P* = 100%, as shown in Figure 3b. Considering that Raman active and structurally disordered areas (normalised per ion impact) are the same, these two cases (shown in Figure 3a,b) cannot be distinguished by Raman spectroscopy. It is also clear that the later result does not conform to *L* = 2 nm requirement. Therefore, it is possible—as shown in this work, to establish the probability of the damage formation *P* by imposing the *L* = 2 nm requirement (Equation (2)) after the analysis of experimental data (i.e., after obtaining r_a_ and r_s_) by the original Lucchese model (Equation (1)).

(3) $$P={\left(\frac{r_{a}-r_{s}}{L}\right)}^{2}$$

Therefore, there is a clear correlation (Equation (3)) between *r_a_* − *r_s_* < 2 nm and the low probability of damage formation *P*. The results of the present analysis, both for BLG and TLG samples, are given in Table 2.

The results presented in Table 2 show that the ion-induced damage does not vary much, since the radius *r_s_* is smaller than one nanometre in practically all cases. This could be related to the fact that the smallest possible damage in graphene, i.e., one vacancy, affects the structure of three hexagonal rings, which should correspond to the area defined by *r_s_* = 0.22 nm. In addition, it is known that Raman spectroscopy tends to overestimate the size of defects (and ion tracks) because the strain fields surrounding defects are also detected [18,38]. If this is the case, then the most varying parameter close to the threshold of damage production is not the damage size itself (i.e., *r_s_*), but the probability of damage production *P* [15,39].

To establish the origin of the damage, the probabilities for damage formation have been correlated with electronic and nuclear energy losses. As shown in Figure 3c, there is a strong linear correlation between the nuclear energy loss (calculated for graphite using the SRIM code [30]) and the probability of damage formation *P*, both for BLG and TLG. On the contrary, there is no such relationship with electronic energy loss, as shown in Figure 3d. In this regard, comparing 23 MeV I, 18 MeV Cu, and 3 MeV Cu data is of special interest, because 23 MeV I and 18 MeV Cu have almost the same electronic energy loss *dE_e_*/*dx*, while 23 MeV I and 3 MeV Cu have almost the same nuclear energy loss *dE_n_*/*dx*. As shown in Table 2, the probability for defect formation both in BLG and TLG is similar for 23 MeV I and 3 MeV Cu, while it is much lower for 18 MeV Cu. Therefore, the response of the BLG and TLG is similar to the irradiation by high-energy heavy ions, with the nuclear energy loss dominating damage formation in both materials. We also note that good linear scaling of damage probability *P* with the nuclear energy loss implies that the energy retention is similar for all used ion beams. 

Finally, we note that for both BLG and TLG, the disordered area appears larger than expected for the 12 MeV Si beams, although the probabilities are vanishing. This beam is the one with the highest ion velocity, and this could be a sign of electronic energy loss contribution because its nuclear energy loss is very small. Still, given the low probabilities of damage production, and the clearly dominant role of nuclear energy loss *dE_n_*/*dx* in damage production by other ion beams, we consider all used ion beams to be below the threshold for ion track formation. This finding is also in agreement with the declared threshold for ion track formation in graphite of 7.3 ± 1.5 keV/nm, although a 100% probability for ion track formation was reported above 18 keV/nm [40,41].

## 4. Conclusions

In the present work, damage formation in BLG and TLG due to high-energy heavy ion impact was investigated. Both BLG and TLG are very promising materials for nanomembrane fabrication (due to graphene’s high mechanical strength and sub-nanometre thickness [3]), and high-energy heavy ion irradiation is a well-established technique for nanomembrane production in other types of materials (most often polymers). By tuning ion irradiation parameters, nanopores of desired sizes can be produced, and thus different applications (such as gas and liquid separation, or even water desalination) can be targeted [3,4,42,43,44,45,46,47].

Raman spectroscopy measurements revealed nanoscale damage when analysed by the Lucchese model, and the low probability of damage formation was correlated with nuclear energy loss. We conclude that the ion irradiation conditions were below the threshold for ion track formation both in BLG and TLG, even for the most energetic 23 MeV I^6+^ beam. The dissipation of deposited energy from the nanometric thin films after the high-energy heavy ion impact makes ion tracks even more difficult to produce [24,28,33,34] and could be strongly contributing to the present study. Therefore, much more energetic heavy ion beams have to be used in applications relying on ion track formation (such as nanomembrane production [3,14]), when the ion track production efficiency should be close to 100%.

## Figures and Tables

**Table 1:** Ion irradiation parameters used in the present study. Calculations were performed using the SRIM code [30] for the graphite target: electronic energy loss *dE*_e_/*dx*, nuclear energy loss *dE_n_*/*d*x, and ion range.

Ion Beam	*dE_e_*/*dx* (keV/nm)	*dE_n_*/*dx* (keV/nm)	Ion Range (μm)
1.8 MeV I^2+^	1.797	1.355	0.55
23 MeV I^6+^	6.724	0.271	5.55
3 MeV Cu^2+^	2.054	0.251	1.7
18 MeV Cu^6+^	6.680	0.007	5.04
12 MeV Si^4+^	4.370	0.001	4.12

**Table 2:** Results of the Raman spectroscopy analysis of irradiated BLG and TLG samples: radius *r_s_* of the structurally disordered area due to the ion impact, radius *r_a_* of the activated area, their difference *r_a_* − *r_s_*, and probability *P* of damage formation due to the ion impact.

	*r_s_* (nm)	*r_a_* (nm)	*r_a_* − *r_s_* (nm)	*P* (%)
**BLG**				
1.8 MeV I^2+^	0.8	1.35	0.55	7.6
23 MeV I^6+^	0.62	1.1	0.48	5.8
3 MeV Cu^2+^	0.7	1.1	0.4	4
18 MeV Cu^6+^	0.66	0.85	0.19	0.9
12 MeV Si^4+^	1.26	1.4	0.14	0.5
**TLG**				
1.8 MeV I^2+^	0.75	1.4	0.65	10.6
23 MeV I^6+^	0.75	1.1	0.35	3.1
3 MeV Cu^2+^	0.65	0.95	0.3	2.3
18 MeV Cu^6+^	0.66	0.85	0.19	0.9
12 MeV Si^4+^	1.2	1.3	0.1	0.3

## Data Availability

Data is available on request.
